# A Worm-Like Biomimetic Crawling Robot Based on Cylindrical Dielectric Elastomer Actuators

**DOI:** 10.3389/frobt.2020.00009

**Published:** 2020-02-11

**Authors:** Sascha Pfeil, Markus Henke, Konrad Katzer, Martina Zimmermann, Gerald Gerlach

**Affiliations:** ^1^Faculty of Electrical and Computer Engineering, Institute of Solid State Electronics, Technische Universität Dresden, Dresden, Germany; ^2^Faculty of Electrical and Computer Engineering, Institute of Semiconductors and Microsystems, Technische Universität Dresden, Dresden, Germany; ^3^PowerOn Ltd., Auckland, New Zealand; ^4^Fraunhofer Institute for Material and Beam Technology IWS, Dresden, Germany; ^5^Faculty of Mechanical Science and Engineering, Institute for Material Science, Technische Universität Dresden, Dresden, Germany

**Keywords:** biomimetics, cylindrical DEA, dielectric elastomer actuators, textile reinforcement, soft robotics, *in-situ* pre-stretch, linear actuators, inflatable actuators

## Abstract

In recent years the field of soft robotics has gained a lot of interest both in academia and industry. In contrast to rigid robots, which are potentially very powerful and precise, soft robots are composed of compliant materials like gels or elastomers (Rich et al., 2018; Majidi, 2019). Their exclusive composition of nearly entirely soft materials offers the potential to extend the use of robotics to fields like healthcare (Burgner-Kahrs et al., 2015; Banerjee et al., 2018) and advance the emerging domain of cooperative human-machine interaction (Asbeck et al., 2014). One material class used frequently in soft robotics as actuators are electroactive polymers (EAPs). Especially dielectric elastomer actuators (DEAs) consisting of a thin elastomer membrane sandwiched between two compliant electrodes offer promising characteristics for actuator drives (Pelrine et al., 2000). Under an applied electric field, the resulting electrostatic pressure leads to a reduction in thickness and an expansion in the free spatial directions. The resulting expansion can reach strain levels of more than 300% (Bar-Cohen, 2004). This paper presents a bioinspired worm-like crawling robot based on DEAs with additional textile reinforcement in its silicone structures. A special focus is set on the developed cylindrical actuator segments that act as linear actuators.

## 1. Introduction

Soft robotics are an emerging field for academic and industrial research. In order to adapt the conventional robotic architecture to changing requirements in both industrial and service robotics, there is a currently change from continuously running assembly lines to more adaptive ones (Zhong et al., 2017). The trend is to develop the handling of one and the same product to more adaptive fabrication techniques that can handle changing products in real-time. Especially in e-commerce applications there is a strong demand for more flexible robots (Liang et al., 2015). The second big driver in industry is the introduction of so-called collaborative robots, or cobots (Romero et al., 2016). In future factories workers and robots will co-exist and collaborate as colleagues. This close collaboration introduces a risk of injuries and accidents caused by the robots. To prevent this and to increase the psychological acceptance of cobots as colleagues, it is advantageous to introduce soft robotic structures where possible.

Before soft, collaborative robotic systems will be widely used, it is necessary to develop materials, processes and components that are soft. Such parts can resemble all sub-components that are necessary to build up conventional robots, such as sensors, actuators, signal processors, and the structure itself. Within the recent years many major breakthroughs on these fields have been made by scientists and engineers all over the world, resulting in the world's first entirely soft, fully functional robot, the octobot, in 2016 (Wehner et al., 2016). Even though the octobot was a world's first, many scientists work on sensors and actuators that are soft and flexible, but can be controlled electrically. Such concepts include electroactive polymers (Bar-Cohen, 2004), especially dielectric elastomers (DEs) (Carpi et al., 2008) or HASEL actuators (Kellaris et al., 2018). DEs have been proven as multi-functional (Anderson et al., 2012) and can be used as signal-generators (O'Brien and Anderson, 2012; Henke et al., 2018) and -processors (Wilson et al., 2016, 2017) for self-controlled, untethered robots (Cao et al., 2017; Henke et al., 2017, 2019). Due to their wide range of applications, there is a focus on electroactive driven soft robotics since end of the 1990s (Pelrine et al., 1998).

Beyond these efforts in the field of soft robotics there is also a trend to make such robots self-sustainable. A core-aspect for self-sustainability is energy autonomy. Different approaches deal with energy harvesting to overcome the need of external power supplies for robotic concepts. Aghakhani and Basdogan (2018) showed a concept to convert mechanical energy into electrical energy by using piezoelectric patches. Also the use of electroactive polymers to generate electrical energy is possible as shown by McKay et al. (2011) or Mathew and Koh (2018). These developments pave the way for fully autonomous robots like the Row-Bot presented by Philamore et al. (2015).

Worm-like robotic concepts based on different soft materials are already implemented. Often these robotic concepts use peristaltic motion with a small number of segments that can contract axially and expand in radial direction (Dario et al., 2004; Omori et al., 2009; Seok et al., 2010). Another approach to achieve forward motion is to use a method of anchored crawling. This method clamps the ends of an actuator element to the ground, which allows the robot to move forward. An actuator element located between the lockable ends ensures the forward motion by expanding in axial direction like in Chowdhury et al. (2017) or Joey et al. (2019). In this paper we present a robot that inherits two features that have been rarely used in the field of soft robotics until now. The first one is the concept to use DEAs in large area complex geometries. In the presented case the actuators are not applied in the well established planar geometry but in a cylindrical shape. This means that the planar DEAs are wrapped around a cylindrical inner holding structure, providing the shape for the actuators. The second innovative feature is to use a textile material as reinforcement structure for the inner silicone parts. Due to miniaturization the used materials become very unstable and fragile. Also the inner structures have to take up some external forces and the forces generated by the DEAs themselves. These forces can possibly lead to undefined deformations and changes of the aspired cylindrical geometry. In the presented concept a textile material consisting of parallel oriented carbon fibers, coated in an styrene-butadiene rubber system is used to ensure the shape consistency. The textile reinforcement enhances the stiffness in the supporting areas of the robot. Due to the reinforcement a miniaturization of the system is possible while the structures still can handle the existing forces without collapsing or breaking. Finally, the movement of the cylindrical DEA elements is limited in a way that they act as linear actuators in length direction. This paper is mainly about the development, description, and the characterization of these cylindrical DEA elements.

## 2. Methods

### 2.1. Robotic Concept

**Figure 1:**
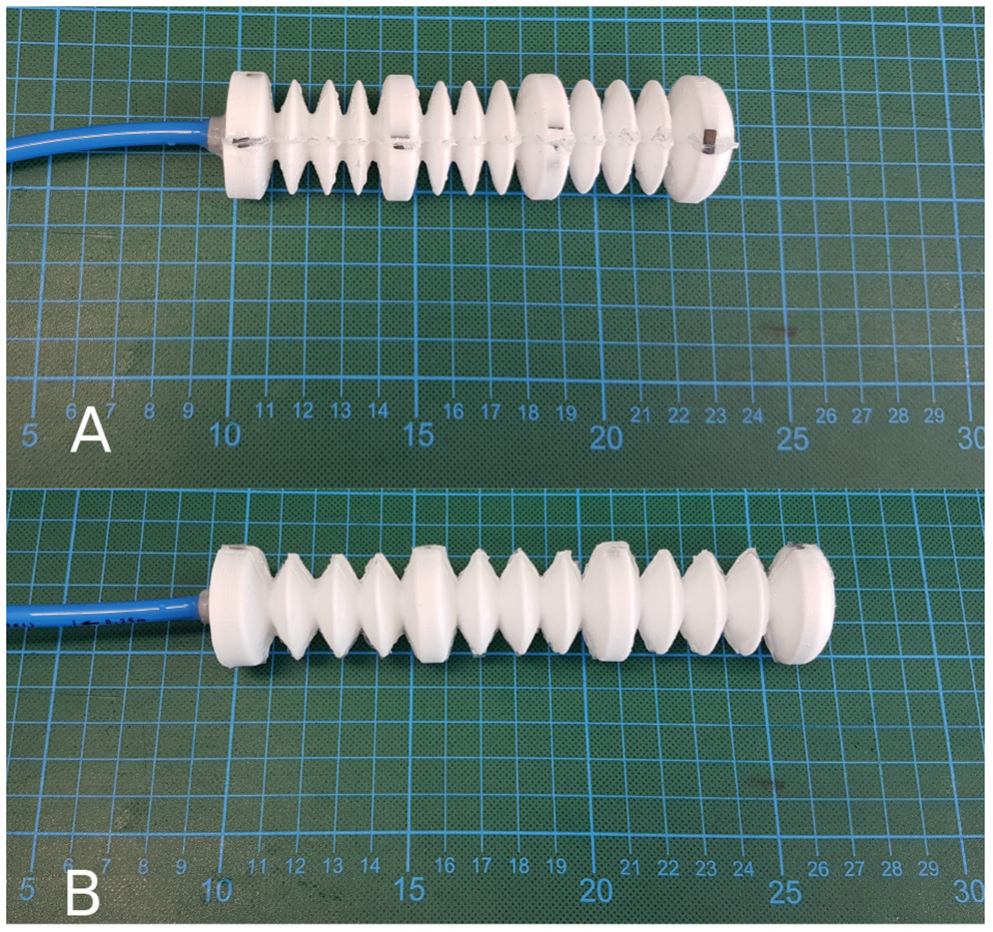
Inner holding structure. **(A)** without applied gas pressure in initial state, **(B)** with an applied gas pressure of 80 mbar.

The aim of the robotic concept is to develop a biomimetic structure that is driven by customized actuator segments of a cylindrical shape. These segments act as the core part of the robot. The whole robotic setup is inspired by the biological role-model of inchworms. They consist (besides many other body parts) of an inner muscular structure with longitudinal oriented muscles and an outer structure with ring muscles, generating the forward movement (Edwards and Bohlen, 1996). In the developed robotic concept the cylindrical actuator segments serve the function of force generation for the movement. The cylindrical actuator segments consist of rolled up DEAs around an inner holding structure. The robot consists of three cylindrical DEAs as actuator segments on the circumference of the inner-holding structure. Their ground electrode is continuously connected to all three actuator elements so it only needs one electrical connection for the ground electrode at the foot-end of the robot. The activation of the single elements then is realized by switching the high voltage electrodes of each actuator element. The inner holding structure is made of a soft silicone mixture *Dow Corning Sylgard 184* © and designed in a way to serve different needs. First, it gives the cylindrical shape to the DEAs. This becomes necessary since the only 100 μm *Elastosil 2030* © dielectric membrane of the DEAs would collapse under its own weight without an additional holding structure. Second, the inner holding structure limits the degrees of freedom for the actuation and ensures a mainly longitudinal expansion. Its geometry is constructed to predominantly elongate in length direction when an internal gas pressure is applied (see Figure 1). Third, it sets the robot to a certain operating point and gives a well defined shape for both the DEAs and the whole setup. In the presented stage of development a pressure pump is used to provide the internal gas pressure. In later developments a pneumatic valve can be used to prevent the compressed air from outflowing. Since the compressed air mainly serves shaping purposes it could also be replaced by another medium. Even approaches toward self-sustainability by eliminating the use of an external pressure pump are possible. As presented by Onal et al. (2017), an integrated pressure generator could be used to provide the internal gas pressure.

**Figure 2:**
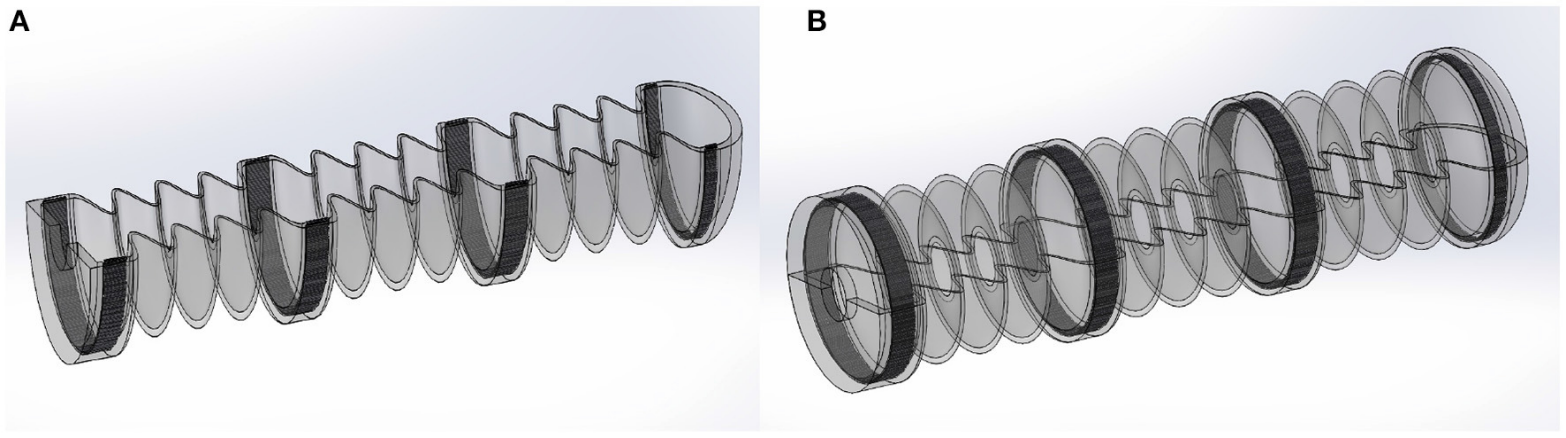
Concept of inner holding structure. **(A)** single half as it is cast with integrated textile material at supporting positions, **(B)** full inner holding structure assembled from two similar cast halfs.

**Figure 3:**
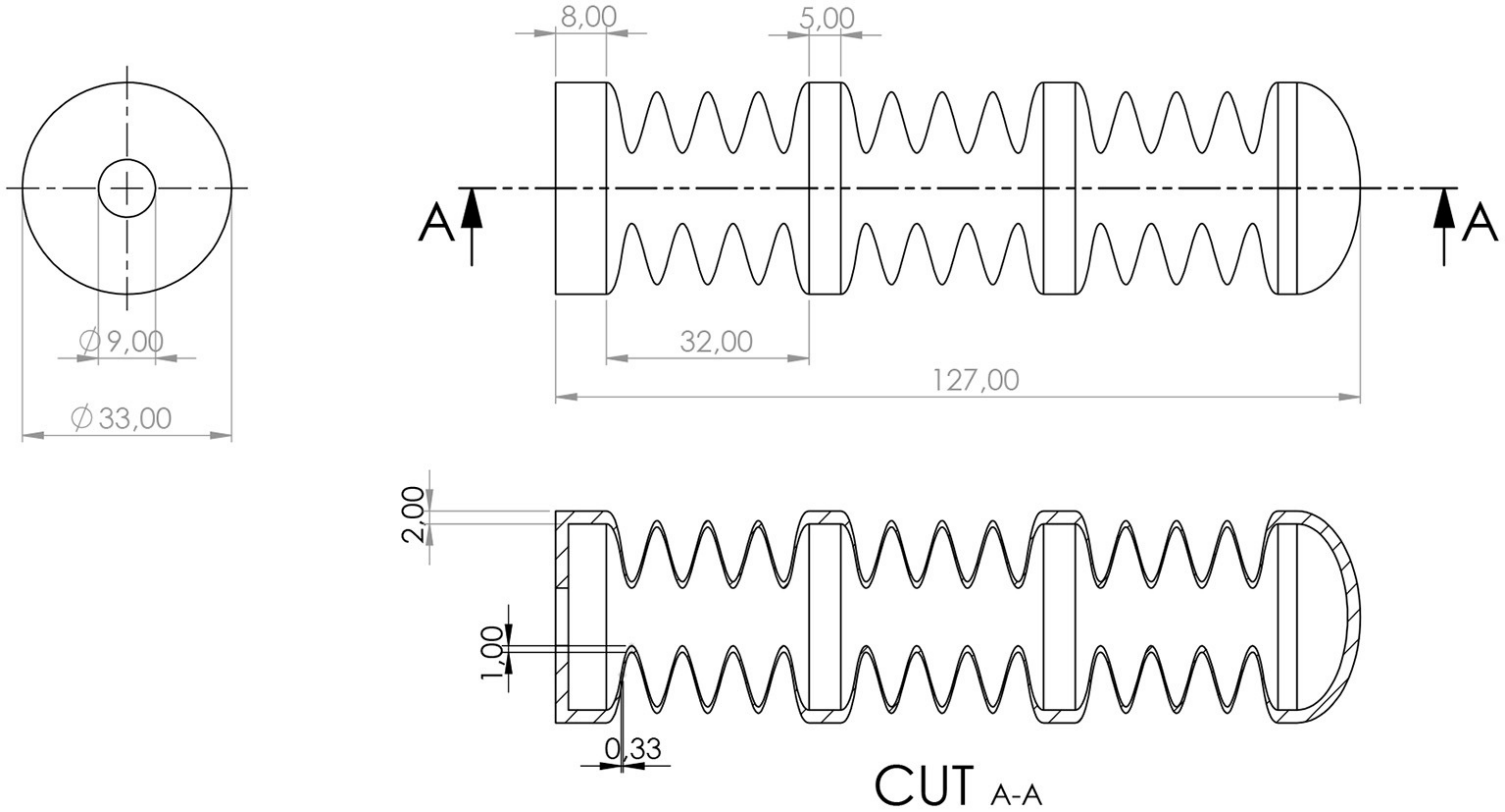
Construction of the inner holding structure with dimensions.

**Figure 4:**
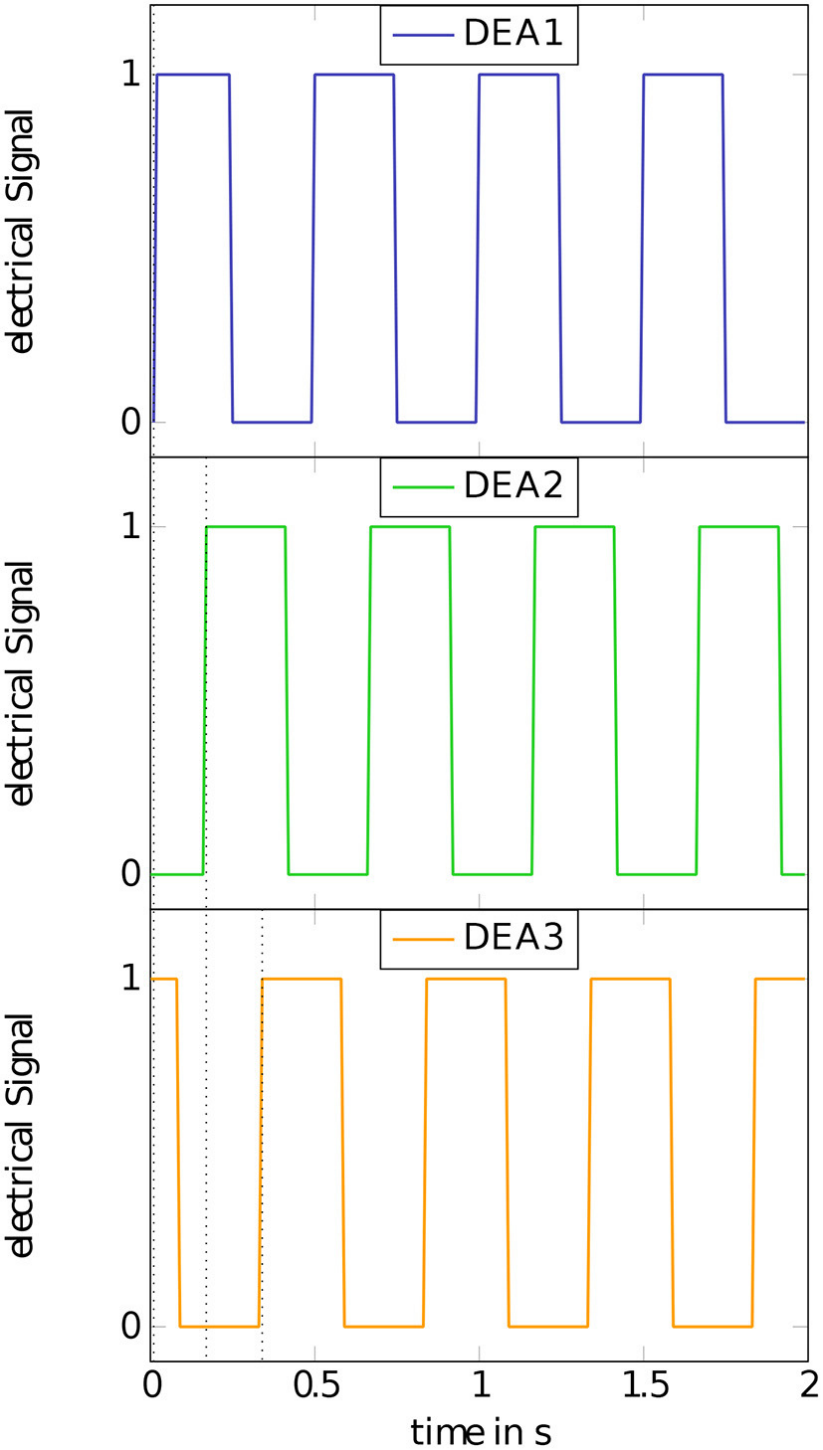
Concept of consecutively switching of the actuator segments. The three DEAs are switched with a phase shift of 120° between each other, performing a square waveform of the voltage.

**Figure 5:**
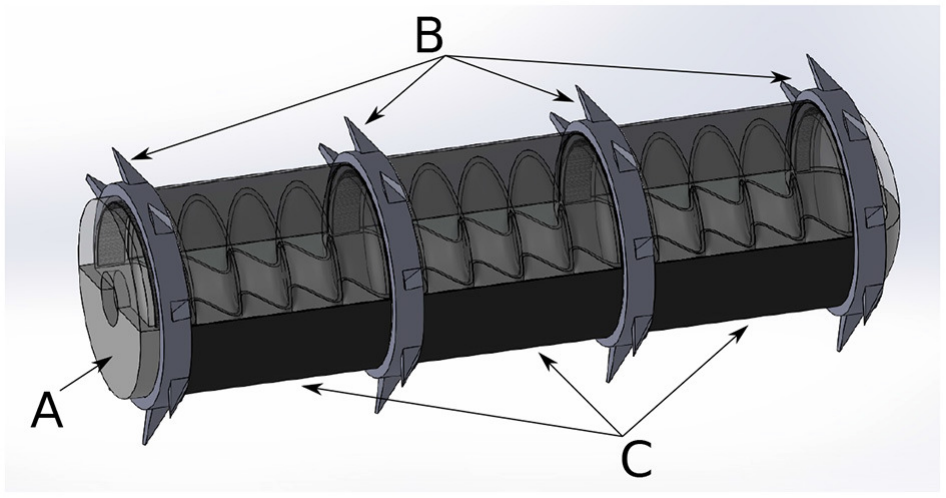
Full robotic concept with, **(A)** inflatable inner holding structure, **(B)** gripping segments with hooks on circumference, and **(C)** cylindrical actuator segments based on dielectric elastomer actuators.

In contrast to established DEA concepts, the developed actuator segments are non-planar and controlled in their movement direction to act as linear actuators. The fabrication of the inner holding structure itself is done in a cast process. Therefore, a cast mold is designed to cast the silicone mixture to a half of the desired geometry. The silicone is cast around a textile material consisting of SGL's *Sigrafil C T50-4.4/255-E100* © fiber with a grammage of 200 $\frac{\text{g}}{{\text{m}}^{2}}$ coated with a low-viscosity *Lefasol VL 90/1* © styrene-butadiene rubber (SBR) system by Lefatex. The textile material is added as an additional stiffening in the supporting rings of the inner structure. It ensures the needed mechanical stability to take up the existing forces due to the application of the additional features for the robotic concept. Figure 2 shows the concept of a single half structure with integrated textile material and the full concept for the inner holding structure consisting of two half structures attached to each other. The dimensions of the inner holding structure are shown in Figure 3. The dimensions are decreased to a point that takes the miniaturization to a limit under common laboratory conditions without special production technologies. Below these dimensions some advanced fabrication technologies would become necessary in order to maintain produceability. The mechanical dimensions of the robot, including its weight and the outer diameter at the tips of the gripper rings are summed up in Table 1. The other functional parts of the robot serve the generation of a forward motion. Namely these are the hook-like gripper rings. Their function is to generate a directional adjusted friction to allow an easy forward movement and to stick to the underlying surface in backward direction. The design of the grippers follows the idea of using hooks to prevent backward movement by anchoring in the ground. The forward movement remains unaffected by this as the geometry allows the anchoring to be released in the forward direction. The movement itself follows the concept of anchored crawling. The three actuator segments are switched with a phase shift of 120° between each other, performing a square waveform of the voltage. Figure 4 illustrates the time behavior of the electrical signals on each DEA for an exemplary switching frequency of 4 Hz. The switching to an electrical active state leads to an expansion of the actuator elements. In the realized concept the actuators elongate with a time overlap between each other. A further function of the gripper rings is to ensure a gap between the outer DEA electrodes and the surface on which the robot is moving in order not to destroy the DEAs. Figure 5 shows the full robotic concept with the previously described elements. The movement itself follows a simple concept of consecutively activating the single actuator segments to generate a forward motion of the robot.

**Table 1:** Overview of the mechanical parameters and values of the robot.

	**Setup value**
Weight	29.08 g
Length	127 mm
Outer diameter	49.5 mm
Gait	Anchored crawling

### 2.2. Theory: Cylindrical Actuation

In order to get an assessment of the generated force that expands the actuator segments of the presented robot, some general electromechanical considerations are made. In comparison to the typical planar DEA setups, the cylindrical geometry of the presented crawling robot requires some considerations adapted to the geometrical conditions. First of all, the use of cylindrical coordinates is advisable to describe the behavior. Besides the change to cylindrical coordinates, some physical assumptions are made. The electrodes on the inner and outer radius are assumed as neglectable thin and perfectly conductive. Also the electrical voltage and therefore the electrical field is assumed as oriented exclusively in radial direction of ${\vec{e}}_{\rho }$ between the electrodes. Due to the use of an inner holding structure the free spatial directions for the expansion of the actuators are limited. While pure cylindrical actuators would mainly expand in radial direction (Carpi et al., 2009), the presented crawling robot expands in longitudinal direction to achieve a forward motion. The limitation of the free spatial directions to expand is ensured by the inflatable inner holding structure. The DEAs are attached to the circumference of the inner holding structure so that the expansion is limited by the mechanical behavior of the inner structure. The inner radius *r*_*i*_ is fixed and does not change. The applied charge Q on the outer electrode leads to an electric field between the electrodes that moves the outer electrode toward the inner electrode. The dielectric material itself is assumed as isotropic and homogeneous. Under these assumptions we derive an expression for the electrical generated maxwell pressure. Using this pressure description as a connection between the electrical and the mechanical behavior, we can describe the generated force of the actuator segments. The generated maxwell pressure on the electrodes can be described using some analytical descriptions for electrodynamics. According to Süße (2006) and Eringen and Maugin (2012), the maxwell stress tensor can be written as:

(1) $$\begin{array}{l} \vec{T}=\vec{E}⊗\vec{E}+\vec{B}⊗\vec{B}-\frac{1}{2}\cdot \epsilon_{0}\left(E^{2}+B^{2}\right) \end{array}$$

which is equivalent to

(2) $$\begin{array}{l} \vec{T}=\epsilon \left(\vec{E}\cdot \vec{E^{T}}-\frac{1}{2}\cdot \text{I}\cdot |E{|}^{2}\right)+\frac{1}{\mu }\left(\vec{B}\cdot \vec{B^{T}}-\frac{1}{2}\cdot \text{I}\cdot |B{|}^{2}\right). \end{array}$$

with the electrical field strength *E*, the dielectric permittivity ϵ, the magnetic field strength *B*, the magnetic permeability μ, and the unit matrix I. Due to the quasi electrostatic case, we can assume the magnetic field strength as zero which simplifies the equation for the maxwell stress tensor to:

(3) $$\begin{array}{l} \vec{T}=\epsilon \left(\vec{E}\cdot \vec{E^{T}}-\frac{1}{2}\cdot \text{I}\cdot |E{|}^{2}\right). \end{array}$$

**Figure 6:**
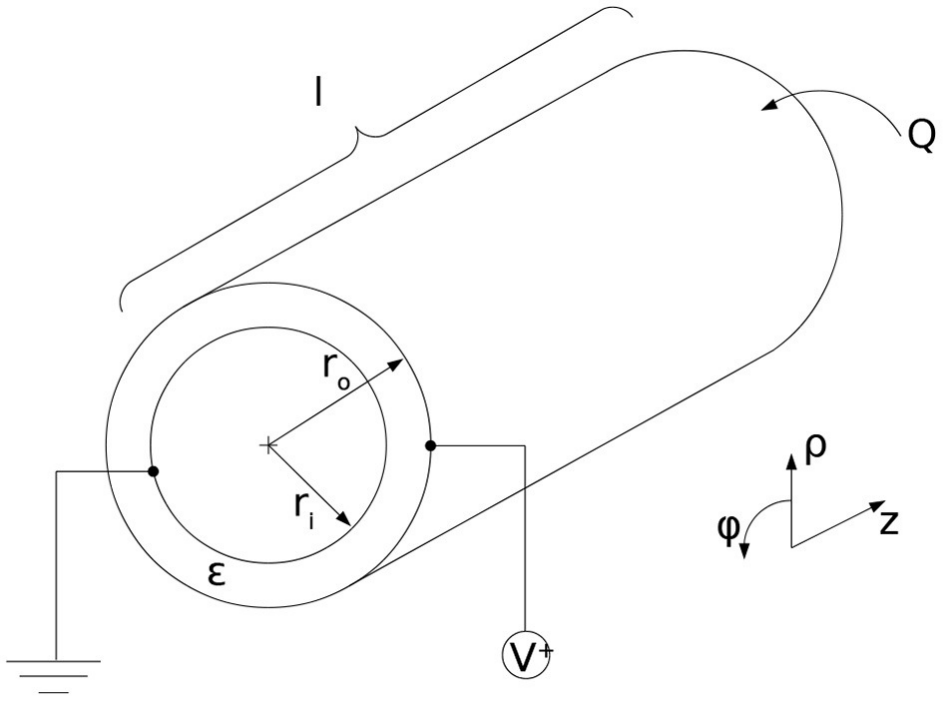
Geometrical conditions for the cylindrical dielectric elastomer actuator with radii r_*i*_ and r_*o*_, length l, applied voltage V and charge Q.

Figure 6 shows the principle geometry conditions for the cylindrical actuator. The first step toward an expression for the maxwell stress is to describe the electric field, caused by the charge distribution σ_*A*_, which contains the charge *Q* over the area of the cylinder wall *A*.

(4) $$\begin{array}{l} \vec{E}=\frac{Q}{\epsilon A}=\frac{\sigma_{A}}{\epsilon }\cdot {\vec{e}}_{\rho ,\phi ,z} \end{array}$$

The derived equation for the electric field can be inserted into Equation (2).

(5) $$\begin{array}{l} \vec{T}=\epsilon \left[\left(\begin{array}{c} E_{\rho }\\ E_{\phi }\\ E_{z} \end{array}\right)\cdot \left(E_{\rho };E_{\phi };E_{z}\right)-\frac{1}{2}\left(\begin{array}{ccc} 1 & 0 & 0\\ 0 & 1 & 0\\ 0 & 0 & 1 \end{array}\right)\cdot E^{2}\right] \end{array}$$

(6) $$\begin{array}{l} \vec{T}=\frac{\epsilon }{2}\left(\begin{array}{ccc} E^{2} & 0 & 0\\ 0 & -E^{2} & 0\\ 0 & 0 & -E^{2} \end{array}\right) \end{array}$$

Using the expression for the maxwell stress tensor, we can calculate the force impact on the cylindrical wall.

(7) $$\vec{F}=\iint_{A}\vec{T}\cdot d\vec{A}$$

The differential area element $d\vec{A}$ comprises of

(8) $$\begin{array}{l} d\vec{A}=r_{\text{o}}d\phi dz\cdot {\vec{e}}_{\rho } \end{array}$$

in cylindrical coordinates. The force can be written as

(9) $$\begin{array}{l} \vec{F}=\frac{\epsilon \cdot \sigma_{A}^{2}}{2\epsilon^{2}}r_{\text{o}}\int_{0}^{l}\int_{0}^{2\pi }d\phi dz. \end{array}$$

After solving the integral, the final expression for the force impact on the cylindric wall is

(10) $$\begin{array}{l} F=\frac{\sigma_{A}^{2}}{2\epsilon }\cdot 2\pi r_{\text{o}}l \end{array}$$

(11) $$\begin{array}{l} F=\frac{\sigma_{A}^{2}}{2\epsilon }\cdot A. \end{array}$$

In order to derive an equation consisting of practical useable values, the expression of the force can be reformulated. To get an expression that depends on the applied voltage *V* instead of the charge distribution σ_*A*_, a consideration of the electric potential Φ in radial direction is necessary. In general, the potential can be expressed as

(12) $$\begin{array}{l} \Phi_{\text{o}}=\Phi_{\text{i}}-\int_{r_{\text{i}}}^{r_{\text{o}}}E\left(r\right)dr. \end{array}$$

With Equation (4) for the electric field from above the potential is

(13) $$\begin{array}{l} \Phi_{\text{o}}=\Phi_{\text{i}}-\frac{Q}{2\epsilon \pi l}\cdot \int_{r_{\text{i}}}^{r_{\text{o}}}\frac{1}{r}dr \end{array}$$

(14) $$\begin{array}{l} \Phi_{\text{o}}=\Phi_{\text{i}}-\frac{Q}{2\epsilon \pi l}\cdot ln\left(\frac{r_{\text{o}}}{r_{\text{i}}}\right). \end{array}$$

Using a description for the applied voltage as a difference of electric potentials

(15) $$\begin{array}{l} V=\Phi_{\text{o}}-\Phi_{\text{i}}=\frac{Q}{2\epsilon \pi l}\cdot ln\left(\frac{r_{\text{i}}}{r_{\text{o}}}\right) \end{array}$$

we can derive an expression for the charge *Q* by converting the equation for the voltage

(16) $$\begin{array}{l} Q=\frac{V2\epsilon \pi l}{ln\left(\frac{r_{\text{i}}}{r_{\text{o}}}\right)}. \end{array}$$

Using this formulation the charge distribution σ_*A*_ can be described as

(17) $$\begin{array}{l} \sigma_{A}=\frac{Q}{A}=\frac{V\cdot 2\epsilon \pi l}{ln\left(\frac{r_{\text{i}}}{r_{\text{o}}}\right)2\pi r_{\text{o}}l}=\frac{V\cdot 2\epsilon }{ln\left(\frac{r_{\text{i}}}{r_{\text{o}}}\right)r_{\text{o}}} \end{array}$$

which leads to an expression for the force of

(18) $$\begin{array}{l} F=\frac{\epsilon V^{2}\pi l}{ln^{2}\left(\frac{r_{\text{i}}}{r_{\text{o}}}\right)r_{\text{o}}}. \end{array}$$

For the further use, we need a description as stress component. Therefore, a division by the area *A* leads to the final expression for the stress component σ_*maxwell*_ in radial direction due to an electric charge on the electrode:

(19) $$\begin{array}{l} \sigma_{\text{maxwell}}=\frac{F}{A}=\frac{\epsilon V^{2}}{ln^{2}\left(\frac{r_{\text{i}}}{r_{\text{o}}}\right)2r_{\text{o}}^{2}}. \end{array}$$

Due to the assumed incompressibility of the silicone material, the derived description for the radial stress component also acts in the other free spatial directions. In this case the maxwell pressure σ_*maxwell*_ also acts on the wall thickness of the cylinder and causes a length dilatation. The area over the wall thickness can be calculated by the following expression.

(20) $$\begin{array}{l} A_{\text{wall}}=\pi \left(r_{\text{o}}^{2}-r_{\text{i}}^{2}\right) \end{array}$$

The force generated by a single actuator segment can than be calculated by the following equation.

(21) $$\begin{array}{l} F=\sigma_{\text{maxwell}}\cdot A_{\text{wall}} \end{array}$$

(22) $$\begin{array}{l} F=\frac{\epsilon V^{2}}{ln^{2}\left(\frac{r_{\text{i}}}{r_{\text{o}}}\right)2r_{\text{o}}^{2}}\cdot \pi \left(r_{\text{o}}^{2}-r_{\text{i}}^{2}\right) \end{array}$$

**Figure 7:**
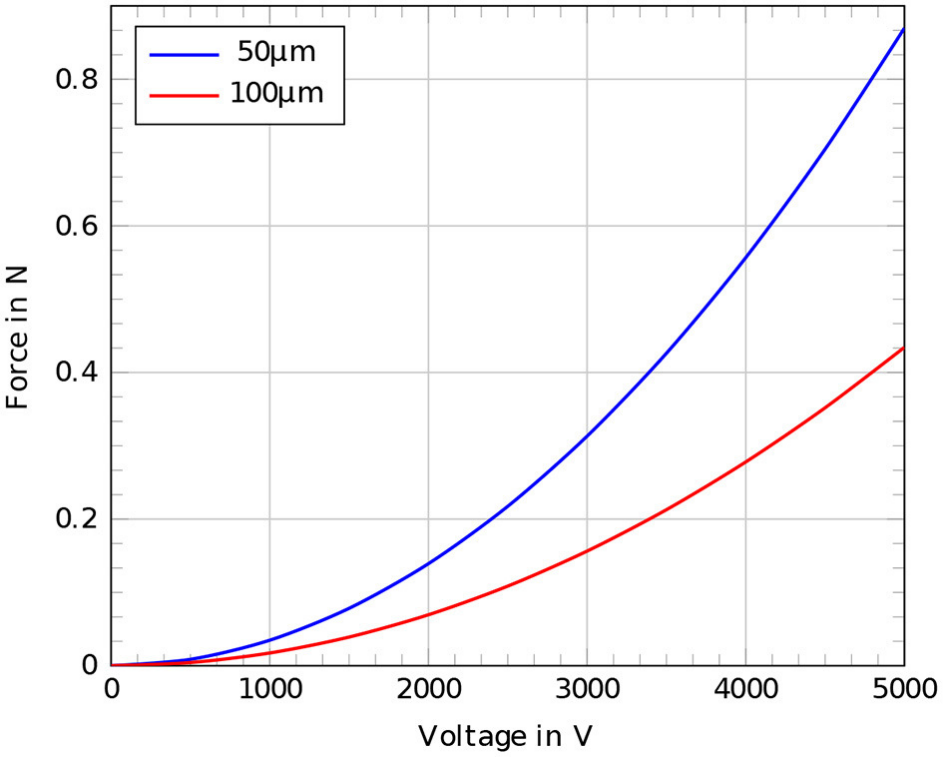
Comparison of the estimated forces according to the analytical derived expression for voltages up to 5,000 V, red: 100μm dielectric membrane, blue: 50 μm dielectric membrane.

For the given dimensions of the crawling robot of *r*_*o*_ = 16.55 mm, *r*_*i*_ = 16.5 mm, and a dielectric permittivity ϵ of $2.8*0.8854*10^{-}11\frac{A\cdot s}{V\cdot m}$ the generated force for an applied voltage of 3, 000*V* equals 0.313 *N*. Figure 7 shows the force curves for different voltages applied to 50 and 100 μm thin dielectric membranes.

## 3. Measurements and Experimental Setup

The characterization of the robotic concept is a crucial task to validate the mechanical behavior of the demonstrator. Therefore, displacement measurements were done to evaluate the extension of the robot. Two demonstrators were used for different test scenarios. To evaluate the behavior of the robotic concept and the single actuator segments, different measurements were performed to characterize the setup. All of the measurements were carried out on a fully built up robot including all the previously described functional parts. For the following measurements two different versions of the robot were used: a version consisting of a 50 μm *Elastosil 2030* © dielectric membrane and a version consisting of a 100 μm *Elastosil 2030* © dielectric membrane. To ensure a well-defined setting of the pressure, a microfluidic pressure pump “Elveflow AF1-P-1600” with a pressure stability of 1 mbar and a power consumption of maximum 15 W according to the datasheet (Elveflow, 2019) was used for all the measurements. The actuators were driven with a high voltage source *Peta-pico-Voltron* delivering output voltages of up to 5 kV and output currents of maximum 220 μA according to Schlatter et al. (2018). Except for the characterization of the crawling movement, a DIC system from GOM, ARAMIS 5M was used to perform camera based displacement measurements. The used camera has a resolution of 2,448 × 2,050 pixels with a focal length of 50 mm. The frame rate was set to a maximum of 15 Hz. After a calibration with a ceramic calibration target, the system was able to detect length differences of a few micrometers. The subset size was set to 19 pixels and the distance to 15 pixels.

### 3.1. Crawling Movement

After building up a demonstrator for pre-testing and characterization of single actuator segments, an adapted full working demonstrator was built. As it turned out, 50 μm thin dielectric membranes in the introduced production method for the DEAs were too fragile to be used. Hence, the membrane thickness was increased to 100 μm. For the testing of the full demonstrator, video captures were taken of the crawling robot acting on 3,000 V supply voltage and 40 mbar inner gas pressure (see Table 2). It turned out that the hook-like ring elements are not sufficient for the crawling on common plane grounds like wood or plastics. Therefore, the ground was slightly tilted by an angle of 1°. At this slightly tilting the robot began to move forward. There was still an observable backwards slippage that was not measured. To not only observe forward gliding due to its own weight no further tilting was applied. To eliminate the influence of the weight force of the feed lines, they were held up using some mountings. Under these conditions the crawling robot got partly enough grip to the surface to move forward. Though the forward movement was not continuous, a few cycles were observed and the displacement was averaged.

**Table 2:** Overview of characteristic parameters and values used for the characterization of the robot.

	**Setup value**
Switching frequency	2 Hz
Voltage	3 kV
Pneumatic pressure	40 mbar
Power consumption	< 3 × 0.66 W

### 3.2. Passive Mechanical Behavior

To evaluate the passive mechanical behavior of the robot, the robotic setup consisting of the more reliably 100 μm dielectric membrane was used. The observed actuator segment was not electrically activated to evaluate the pure passive elongational behavior for different internal gas pressures *p*_gas_. The pressure values were set from 0 to 110 mbar in 10 mbar steps. For each pressure value the displacement was captured to receive the pressure-dependent elongational behavior of a single actuator segment.

### 3.3. Elongation Measurement

**Figure 8:**
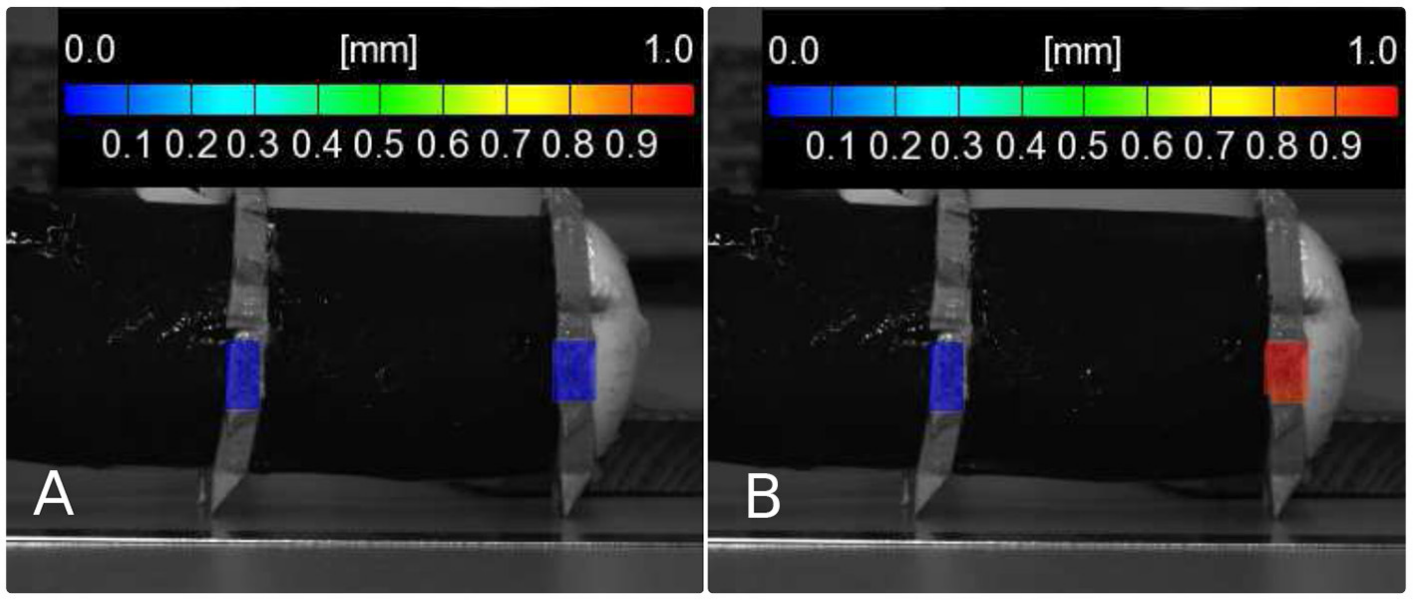
Image for the DIC measurement of the single actuator segment with an applied gas pressure of 60 mbar, **(A)** in the initial state, **(B)** with an applied voltage of 3,500 V.

Despite the deficient suitability of the robotic setup with 50 μm thin dielectric membranes for a fully operational concept, displacement measurements for a single actuator segment were performed on this structure. The images were captured from a distance of about 1 m to the demonstrator. Marker points with a speckle pattern for the DIC-measurement were adhered to the circumference of the hook-like gripping segments of the robot. To evaluate the elongation of a single actuator segment, the front end segment of the robot was chosen for displacement measurements. The robot was introduced to air pressures at 20, 40, 60, and 80 mbar. Each pressure setting was tested at different voltages of 2,000, 3,000, and 3,500 V. The displacement was captured for every single operating point using the DIC system. Figure 8 shows two example images for the described measurement in the initial and in the activated state.

### 3.4. Force Detection

**Figure 9:**
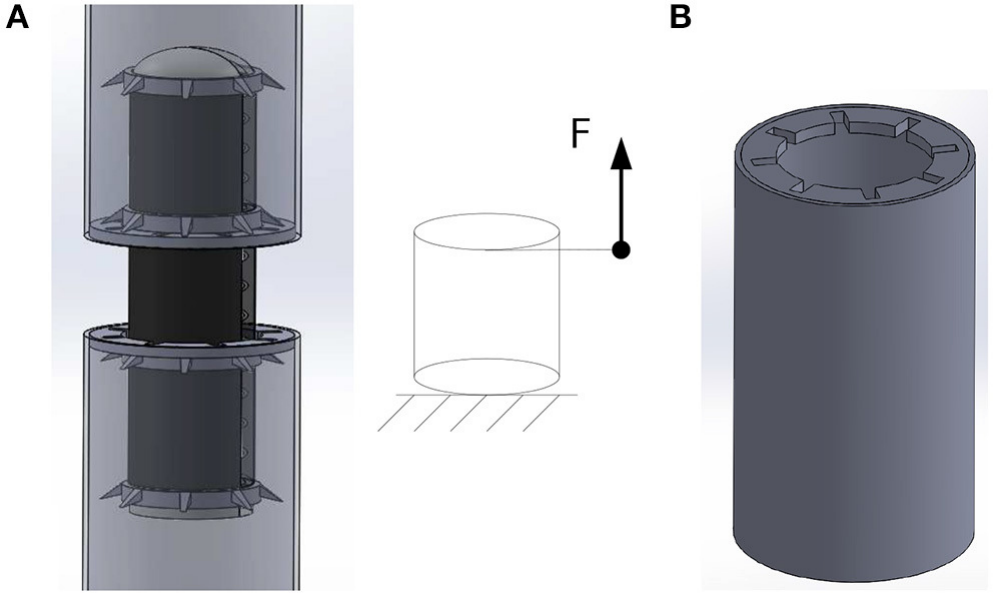
Concept for the force measurement. **(A)** Measurement setup with applied forces to pre-stretch to a working point. **(B)** Construction to take up one end of the cylindrical actuator segment and lock in the hooks to enable an exclusively linear measurement without angular displacement.

Regardless of the previous experiments, additional measurements were done concerning the voltage-dependent force generated by a cylindrical DEA to expand a segment. The force measurements were performed on the previously mentioned fully working robotic setup with a 100 μm dielectric membrane. These measurements were performed on a Zwick-Roell Z050 tensile testing machine with a force transducer of F_*max*_ = 100 N. To take up the robot and to measure the generated force of a single actuator segment, some customized mountings were fabricated. These mountings ensure to take up the generated force along the whole circumference of the ring muscle and to translate them in a linear direction to the force transducer without an angular displacement. The worm-like robot is fixed in two of these mountings and slightly pre-stretched. Figure 9 shows the general measurement setup for the force measurement and the concept of the customized mountings.

To measure exclusively the generated force without any impact of the weight of the robot or geometrical variations, the setup is pre-loaded with a force *F*_pre_ of 1.89 N. In this state the setup is fixed in the tensile testing machine on a defined position and can not move beyond that position. In the case of the performed measurements the defined position was at an elongation of 4.776 mm in length direction. The activation of the clamped actuator segment leads to a decreased measured force *F*_*m*_ because of the limited freedom of movement. The generated force *F* can be described as:

(23) $$\begin{array}{l} F=F_{\text{pre}}-F_{m} \end{array}$$

## 4. Results and Discussion

The full robotic concept consisting of a 100 μm dielectric membrane was not performing a continuous forward motion with enough generated grip on the hook-like grippers. Therefore, only an averaged measurement of the forward motion was performed. At a switching frequency of 2 Hz with an applied voltage of 3,000 V the robot moved 7 mm in forward direction after 30 cycles, meaning a displacement of 0.23 mm per switching cycle and a velocity of 28 $\frac{\text{mm}}{\text{min}}$.

**Figure 10:**
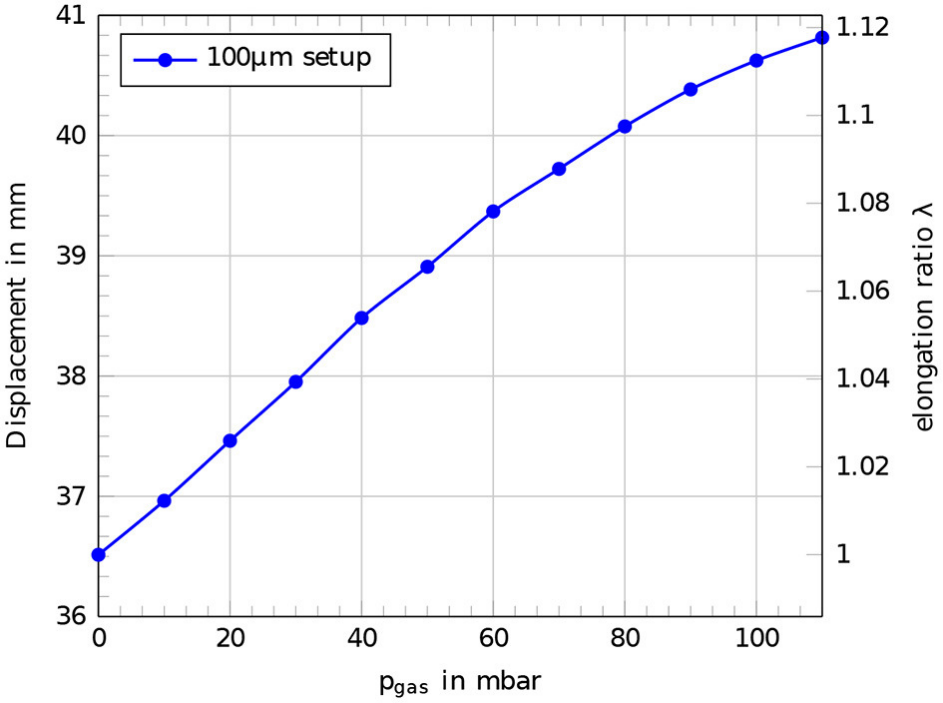
Passive mechanical behavior of a single actuator segment of the robotic concept with a 100 μm dielectric membrane. Displacement and elongation ratio λ for different applied internal gas pressures *p*_gas_.

The passive mechanical behavior of a single cylindrical actuator segment followed a linear behavior for lower internal gas pressures *p*_gas_ up to 60 mbar. Beyond 60 mbar the structure became stiffer in a way that the expansion for a further increasing of the internal gas pressure became lower and the displacement curve flattened in higher pressure ranges. Figure 10 shows the displacement curve as a function of the internal gas pressure *p*_gas_ for a robotic setup with a 100 μm dielectric membrane.

**Figure 11:**
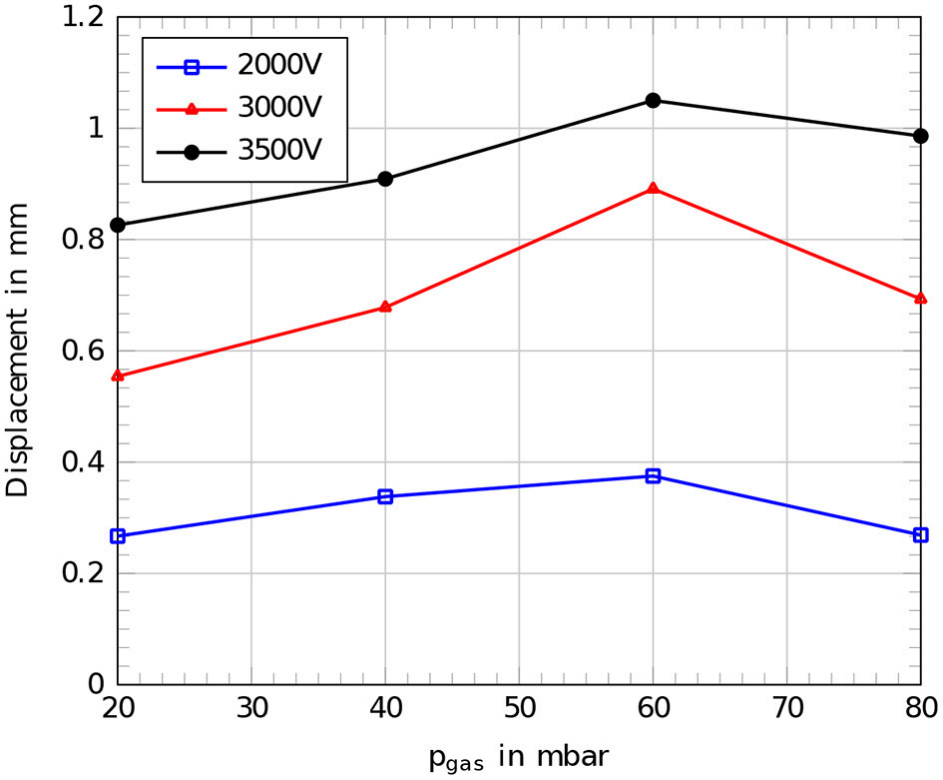
Comparison of measured displacements for the setup with a 50 μm dielectric membrane at different internal gas pressures *p*_gas_ for different applied voltages, blue: 2,000 V, red: 3,000 V, black: 3,500 V.

The measurements for the active behavior of a single cylindrical actuator segment were performed on a robotic setup with 50 μm dielectric membranes. By electrical activation of the cylindrical DEAs, the displacement is increasing in every case with an increasing voltage on the electrodes. Also an increasing internal gas pressure *p*_gas_ leads to an increasing displacement until a point where the whole structure becomes stiffer. The optimum operation point can be assumed in the range around 60 mbar. Beyond this pressure the whole structure becomes stiffer and the actuation is reduced. A maximum deflection of 1.05 mm was recorded for an internal gas pressure of 60 mbar and an applied voltage of 3,500 V on the electrodes of the cylindrical DEA. Figure 11 shows the results of the measurements for the active elongation behavior.

**Figure 12:**
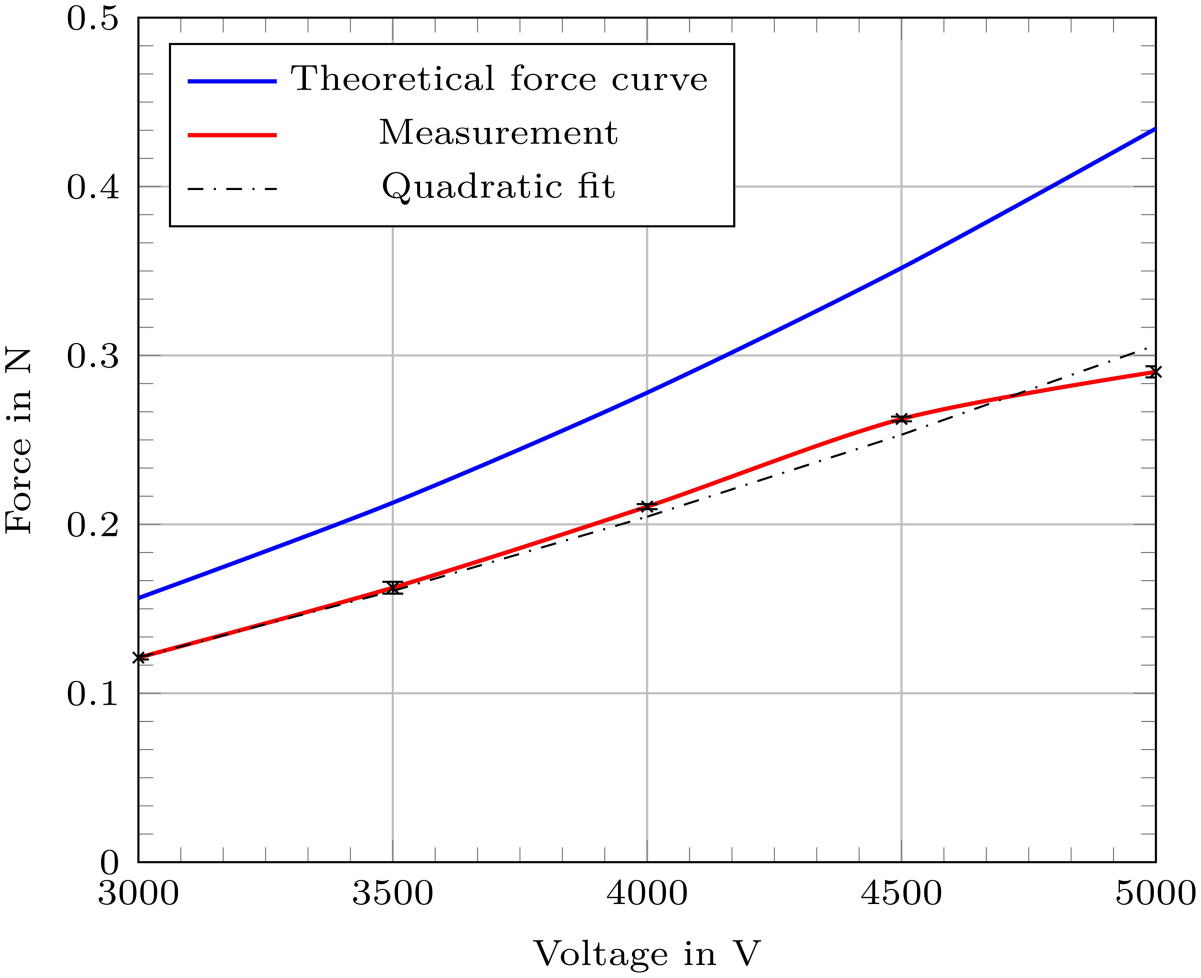
Generated force of a single actuator segment of the robotic concept with a 100 μm dielectric membrane with comparison to the theoretical values (blue) and an additional quadratic fitting of the measured values.

The force measurements were performed on a robotic setup consisting of a 100 μm dielectric membrane. The results followed the expected values from the theoretical calculation with a maximum offset of 0.144 N. Also a quadratic fitting between the measured values resulted in good conformity to the expected curve from the theoretical calculation. A generated maximum of 0.29 N was detected for an applied voltage of 5,000 V. The applied pre-loading force *F*_*pre*_ of 1.89 N led to an elongation of 4.7 mm of the measured actuator segment which corresponds to an internal gas pressure of 100 mbar. Figure 12 shows the results of the force measurement with a comparison to the expected values from theory and a quadratic fit of the measured values. The offset to the theoretical force curve can be explained by the imperfection of the fabricated cylindrical actuator segments. The DEAs can not be assumed as perfectly cylindrical since they are built by wrapping the dielectric membrane around the inner structure. This fabrication method inevitably leads to a seam where the edges of the membrane need to overlap. To avoid electrical shortcuts and to increase reliability, there was no coating of an outer electrode attached in the area above the seam. Both features, the non-fully cylindrical coating of an outer electrode and the overlap of the dielectric membrane lead to deviations from the expected behavior.

## 5. Conclusion

The presented robotic concept shows sufficient capabilities of expanding cylindrical actuator elements based on DEAs as robotic drives and to use them as linear actuators. The direction of expansion is successfully limited in longitudinal direction by attaching the cylindrical DEAs to the circumference of an inner holding structure. In the presented implementation and geometry, relative elongations of up to 2.4 % and generated forces of 0.29 N were achieved. The achieved velocity of 28 $\frac{\text{mm}}{\text{min}}$ is not optimal since backward slippage occurred. By optimizing the gripper design the velocity could easily be improved. As shown in the theoretical considerations, the use of thinner dielectric membranes leads to higher generated forces at similar operating voltages. Additionally, thinner membranes lead to a softer expected mechanical behavior of the setup, which leads to higher expected elongations. Therefore, it seems to be advisable to use as thin dielectric membranes as possible. The proceedings of this work showed that the use of thinner dielectric membranes to produce cylindrical DEAs however leads to some additional challenges. In case of the manufacturing and handling of the robotic setup with common laboratory equipment, soon a limit of handling capability is reached. The used 50 μm dielectric membranes tend to develop cracks more easily than the later used 100 μm dielectric membranes. Also the positioning and the application itself is far more challenging for thinner membranes. A main source for possible flaws is generated through the manufacturing process itself. The implemented fabrication strategy leads to an overlap of the membrane that stiffens the actuator segment along the seam. Also the area directly above the seam could not be coated with an electrode to prevent electrical shortcuts between the electrodes. A possible way to overcome these effects is to produce a dielectric membrane in the form of a sleeve. The sleeve geometry would avoid overlap and mechanical in homogenities. Some preliminary experiments on fabricating a dielectric membrane in such a fully cylindrical geometry were done. Therefore, a silicone mixture *Dow Corning Sylgard 184* © was put into a rotating heated rod and cured under constant rotation. Afterwards the cured sleeve was released from the rod for further processing. Because of some restrictions according the handling of such a sleeve no demonstrator was built using this technique. The sleeve tended to collapse during handling and to stick to itself. Also a further application of electrodes would be by far more challenging since the sleeve has to be turned inside out after one electrode is applied to manufacture the other electrode. Besides the challenges the use of sleeve dielectric membranes is definitely an advisable approach to improve the concept of the introduced cylindrical linear actuators for future developments. Beyond possible improved fabrication methods, the operating point for the linear actuators should be set to its optimum. As the results for the elongation under electrical activation suggest, there is an optimal operating point at a certain internal gas pressure. To find this optimum point, some measurements of the passive mechanical behavior should be done under a higher resolution. With more substeps for the applied internal gas pressure, the point of decreasing slope can be narrowed. Afterwards the electrical activated measurements can be performed around this point to find an optimum operating point for a maximum elongation. The presented concept of cylindrical linear actuators based on DEAs shows promising capabilities to act as driving elements for soft robotics. A wide range of applications is possible since the mechanical properties of the used materials fit for a lot of applications where rigid robotics are self-excluding. Therefore, fields like aerospace engineering or human-machine interaction in health care emerge beyond the presented use as a drive for soft robotic concepts.

## Data Availability Statement

All datasets generated for this study are included in the article/supplementary material.

## Author Contributions

SP contributed the majority of the text, developed the constructions, built up the demonstrators, and did the theoretical calculations. MH contributed support for the conception, the layout for the soft structures, and provided help with the experimental setups. KK contributed the majority of the measurements and did the conception for the measurements in accordance with SP and also contributed main parts for the text according the measurements. MZ and GG contributed scientific support and coordination as well as recommendations for the typesetting.

### Conflict of Interest

MH was employed by the company PowerOn Ltd. The remaining authors declare that the research was conducted in the absence of any commercial or financial relationships that could be construed as a potential conflict of interest.
